# 74 Patient’s Perception of Likelihood to Attend Follow-Up in Burn Surgery Clinic

**DOI:** 10.1093/jbcr/irae036.066

**Published:** 2024-04-17

**Authors:** Alexandra Coward, Megan Gilbert, Anastasiya Ivanko, Jason Heard, Soman Sen, Tina L Palmieri, David G Greenhalgh, Kathleen S Romanowski

**Affiliations:** UC Davis, Sacramento, CA; University Medical Center (LSU Health), New Orleans, LA; University of California - Davis Hosp, Sacramento, CA; UC Davis, Shriners Children's Northern California, Sacramento, CA; Shriners Hospital Northern California, Sacramento, CA; UC Davis and Shriners Children's Northern California, Sacramento, CA; UC Davis, Sacramento, CA; University Medical Center (LSU Health), New Orleans, LA; University of California - Davis Hosp, Sacramento, CA; UC Davis, Shriners Children's Northern California, Sacramento, CA; Shriners Hospital Northern California, Sacramento, CA; UC Davis and Shriners Children's Northern California, Sacramento, CA; UC Davis, Sacramento, CA; University Medical Center (LSU Health), New Orleans, LA; University of California - Davis Hosp, Sacramento, CA; UC Davis, Shriners Children's Northern California, Sacramento, CA; Shriners Hospital Northern California, Sacramento, CA; UC Davis and Shriners Children's Northern California, Sacramento, CA; UC Davis, Sacramento, CA; University Medical Center (LSU Health), New Orleans, LA; University of California - Davis Hosp, Sacramento, CA; UC Davis, Shriners Children's Northern California, Sacramento, CA; Shriners Hospital Northern California, Sacramento, CA; UC Davis and Shriners Children's Northern California, Sacramento, CA; UC Davis, Sacramento, CA; University Medical Center (LSU Health), New Orleans, LA; University of California - Davis Hosp, Sacramento, CA; UC Davis, Shriners Children's Northern California, Sacramento, CA; Shriners Hospital Northern California, Sacramento, CA; UC Davis and Shriners Children's Northern California, Sacramento, CA; UC Davis, Sacramento, CA; University Medical Center (LSU Health), New Orleans, LA; University of California - Davis Hosp, Sacramento, CA; UC Davis, Shriners Children's Northern California, Sacramento, CA; Shriners Hospital Northern California, Sacramento, CA; UC Davis and Shriners Children's Northern California, Sacramento, CA; UC Davis, Sacramento, CA; University Medical Center (LSU Health), New Orleans, LA; University of California - Davis Hosp, Sacramento, CA; UC Davis, Shriners Children's Northern California, Sacramento, CA; Shriners Hospital Northern California, Sacramento, CA; UC Davis and Shriners Children's Northern California, Sacramento, CA; UC Davis, Sacramento, CA; University Medical Center (LSU Health), New Orleans, LA; University of California - Davis Hosp, Sacramento, CA; UC Davis, Shriners Children's Northern California, Sacramento, CA; Shriners Hospital Northern California, Sacramento, CA; UC Davis and Shriners Children's Northern California, Sacramento, CA

## Abstract

**Introduction:**

Previous work has demonstrated that follow-up rates in burn patients are concerningly low. The ability to predict which patients will follow up in the clinic could improve short and long-term patient outcomes; through providing alternative means to obtain care. This study aims to evaluate if patients are aware of their likelihood of attending follow-up appointments.

**Methods:**

Following the determination by the IRB that this was an exempt project and did not require informed consent, a survey assessing patient’s confidence that they would attend follow-up, was obtained for all admitted Burn Surgery patients at the time of discharge from September 2021 to June 2022 (N=251). Chart review then determined if patients presented for follow-up within thirty days of discharge. Univariate statistical analysis was performed. For all statistically significant variables, multivariate logistic regression with backward elimination was performed.

**Results:**

Of the 251 included patients, 65% (N=165) presented for follow-up. These patients were able to accurately predict if they would present to follow-up (p < 0.0001). Of patients who were either very or somewhat confident that they would follow up (217 patients), 151 (69.6%) did attend a follow-up. Of patients who were either very or somewhat unconfident (8 patients), 5 patients (62.5%) did not attend a follow-up. A history of substance use disorder (p=0.004), tobacco use (p=0.0004), housing insecurity (p < 0.0001), disposition to a location other than their prior residence (p=0.02), and MediCal or Medicare insurance (p=0.003) were associated with lack of follow up care. Factors independently associated with follow-up include confidence in presenting on survey Odds Ratio (OR)=1.36 (95% Confidence Interval (CI) 0.18-9.8), no housing insecurity OR=4.17 (95% CI 1.53-11.32), and no history of substance use disorder OR=3.09 (95% CI 1.47-6.49).

**Conclusions:**

Patients can accurately predict if they will follow up after discharge, while housing insecurity and history of substance use disorder are independently associated with a lack of follow-up care. Further research on alternatives to traditional follow-up is warranted to improve patient outcomes.

**Applicability of Research to Practice:**

Alternative methods of follow-up should be considered for patients who do not think they will be able to follow up in burn clinic.

